# Evolution in the treatment of multiple myeloma and impact on dialysis independence: data from a French cohort from 1999 to 2014

**DOI:** 10.1038/bcj.2016.17

**Published:** 2016-03-25

**Authors:** M Laforet, N Jourde-Chiche, F Haddad, M Sallee, A M Stoppa, P Brunet, B Dussol, S Burtey, B Gondouin

**Affiliations:** 1 Centre de Nephrologie et Transplantation Rénale, Hopital La Conception, Marseille, France; 2 Vascular Research Center of Marseille, Faculté de Pharmacie, Aix-Marseille Université, Marseille, France; 3 Department of Hematology, Institut Paoli Calmette, Marseille, France

Renal impairment is a frequent complication in patients with multiple myeloma (MM) with an important impact on survival.^1, 2^ It is found in 20–40% of cases at diagnosis and in almost 50% in the disease course. Ten percent of MM patients will require renal replacement therapy support.^3^ A poor prognosis seems to be associated with a glomerular filtrate rate (GFR) of <30 ml/min and/or the need for dialysis.^2^ Renal impairment can be reversible although in patients with very low GFR.^4^ Response to chemotherapy (CT) is necessary to obtain renal response. The complete recovery of renal function is associated with a better overall survival.^5^ Also, the achievement of dialysis independence is linked to a better survival.^6^

During the past decade, the management of myeloma has evolved. Several studies have reported an improved disease-free survival with the advent of new first line CT protocols such as bortezomib. Moreover, bortezomib used in combination with melphalan and prednisone as a first line CT leads to a shorter renal recovery when compared with melphalan plus prednisone.^7^ Dimopoulos *et al.*^8^ have shown an increased survival in patients with renal involvement with the advent of novel therapies, but included only a few dialysis-dependent patients. So far, only few studies have focused on the outcome of patients requiring dialysis when comparing the recent CT options versus previous ones. We performed a retrospective analysis over the past 15 years of patients admitted to our Nephrology Unit with renal impairment and diagnosis of MM. We aimed to analyze the impact of the change in use of CT protocols over the years on dialysis independence achievement, overall survival and renal recovery.

We performed a retrospective analysis on our in-center medical records based on the CIM-10 classification. We found 135 patients admitted to our Nephrology unit between 1999 and 2014 with the diagnosis of MM and acute renal failure.

Patients were included in the analysis if they responded to the following criteria: age between 18 and 90, diagnosis of MM according to the International Myeloma Working Group criteria and renal involvement (RI) defined by a rise in creatinine above 177 μmol/l (>2 mg/dl),^9^ absence of amyloid light-chain amyloidosis or significant albuminuria, a previous normal renal function or chronic kidney disease from another cause. We included patients at diagnosis of MM and patients at relapse with no previous renal involvement of MM.

Patients were divided in two groups based on the availability of novel agents in France. More specifically because bortezomib became available as a first line therapy in 2007, we separated patients in two groups representing two periods of time: those admitted between ‘1999 and 2007' and between ‘2008 and 2014'.

We defined the dialysis independence by the time the patient does not need chronic dialysis anymore. We collected the survival data defined by myeloma-related death and death from any cause. Complete renal recovery was defined as a decrease of serum creatinine below 177 μmol/l (2 mg/dl) after the beginning of myeloma treatment.

Comparisons between groups for numerical variables were performed by the Mann–Whitney *U* test. Differences were considered significant when *P* was <0.05. Comparisons for categorical variables among groups were made using the *χ*^2^ test and Fisher's exact test when appropriate. Time-to-event curves for survival were plotted according to the Kaplan–Meier method and comparisons among groups were performed using the log-rank test. Statistical analysis was performed with the Prism software (GraphPad Software Inc, San Diego, CA, USA).

Eighty-eight patients were analyzed in the ‘1999–2007' period (first period) and 47 patients were analyzed in the ‘2008–2014' period (second period). Patient's characteristics are detailed in Table 1. We did not find any significant differences between the two periods in terms of age, sex ratio and creatinine level at diagnosis of RI. Sixty-nine (69%) patients were dialysis-dependent at hospital admission in the ‘1999–2007' period compared with 25 (53%) in the ‘2008–2014' period (*P*<0.05). Nine patients (10%) had a relapsing myeloma at the time of RI in the first period compared with 10 (21%) in the second period (*P*<0.05). All the other patients had a renal involvement at myeloma diagnosis. Patients in the ‘1999–2007' period had a significantly lower Bence Jones proteinuria (*P*<0.05) and a lower calcemia (*P*<0.05).

CT protocols usage is detailed in Supplementary Table 1. In the ‘1999–2007' period, only five patients (6%) received bortezomib-containing regimens. In the ‘2008–2014' period, patients mostly received bortezomib-containing protocols (35 patients (74%)).

Sixty-one patients (69%) required dialysis compared with 25 patients (53%) in the ‘1999–2007' and ‘2008–2014' period, respectively. Among those patients, at 30 days, 56 patients (92%) remained dialysis-dependent compared with 20 (80%) in the first and second period, respectively. At 6 months, 55 patients (91%) remained dialysis-dependent compared with 15 (60%) in the first and second period, respectively. At 2 years after initial admission, 51 patients (83%) remained dialysis-dependent compared with 14 patients (56%) (Supplementary Table 2).

Kaplan–Meier analysis shows an improvement in dialysis independence in the second period compared with the first (*χ*^2^=7.91, *P*<0.01) (Figure 1).

We then compared patients who received bortezomib with those who received other regimens. Among the 86 patients who required dialysis at admission, 33 (38%) received bortezomib in their CT protocol and 53 (62%) of them received in other protocols. Kaplan–Meier analysis shows an improvement in dialysis independence when bortezomib regimens were used compared with others (*χ*^2^=4.40, *P*<0.05) (Supplementary Figure 1).

During the follow-up, Kaplan–Meier analysis did not show any differences in the overall survival in complete renal response when comparing the two periods (Supplementary Figures 2 and 3).

Renal impairment is a well-known risk factor for shorter survival in patients with MM.^2^ It is believed that renal insufficiency can be reversible in about three quarters of patients with mild-to-severe renal failure, but it is not obvious for the patients needing dialysis support. In our study, we show a benefit over time with the use of novel therapies on dialysis independence when patients needed dialysis at admission. After 2 years of follow-up, 83% of patients remained dialysis-dependent in the ‘1999–2007' period versus 56% in the ‘2008–2014' period. Our study shows a significant improvement in dialysis independence over time, probably related to the widespread use of novel molecules, mainly bortezomib. So far, few studies have focused on MM patients with dialysis support requirement. Our team has already shown an improvement on survival and dialysis independence in myeloma and amyloid light-chain amyloidosis over the years.^10^ Haynes *et al.*^6^ showed that dialysis independence was independently associated with survival over a 20- year period. Nevertheless, they did not find any differences in survival when comparing the two different decades. Another recent study showed that independence of dialysis was associated with survival and lower ß_2_-microglobulin levels were associated with renal recovery.^11^

We did not find any improvement in survival with the use of novel therapies in CT protocols. Dimopoulos *et al.*^8^ showed an improved survival in patients with MM and severe RI treated with novel agents. There are differences between this study and ours. This study included only 3–5% of patients necessitating dialysis at the time of diagnosis. Our study focused on even more severe presentations with almost 50% of patients necessitating dialysis at hospital admission. Moreover, in our study, the high number of patients on hematological relapse in the ‘2008–2014' period may have had an impact on survival analysis. Relapse is of poor prognosis in MM.^12^ Maybe if the relapse rate in the two groups had been comparable, we could have found an improvement in survival in the most recent period as in the study by Dimolpoulos *et al.*^8^

Our study has several limitations. First, our collected data does not allow us to affirm that renal involvement in all the patients is due to myeloma kidney. Cases of renal failure related to dehydration or drug toxicity could have been recorded and analyzed in our cohort. Also, there are only few data available in our cohort on renal histology. Moreover, the type of dialysis modality was not recorded. Second, we analyzed concomitantly patients at diagnosis of MM and patients at relapse. Those two populations have different hematological and renal response rates.^13^ Finally, plasma concentration and bioavailability of CT drugs could have been modified by dialysis itself.^12^

Studies focusing on myeloma patients requiring dialysis are now needed to determine the best treatment options. On this topic, the results of two multicenter studies are awaited.^14, 15^

In conclusion, our study shows that, in myeloma patients with severe renal involvement, active therapy leads to a better dialysis independence achievement. Whether this increase in dialysis-free survival leads to improvement in survival remains to be demonstrated in this subgroup of patients and in the myeloma patients requiring dialysis in the course of the disease.

## Figures and Tables

**Figure 1:**
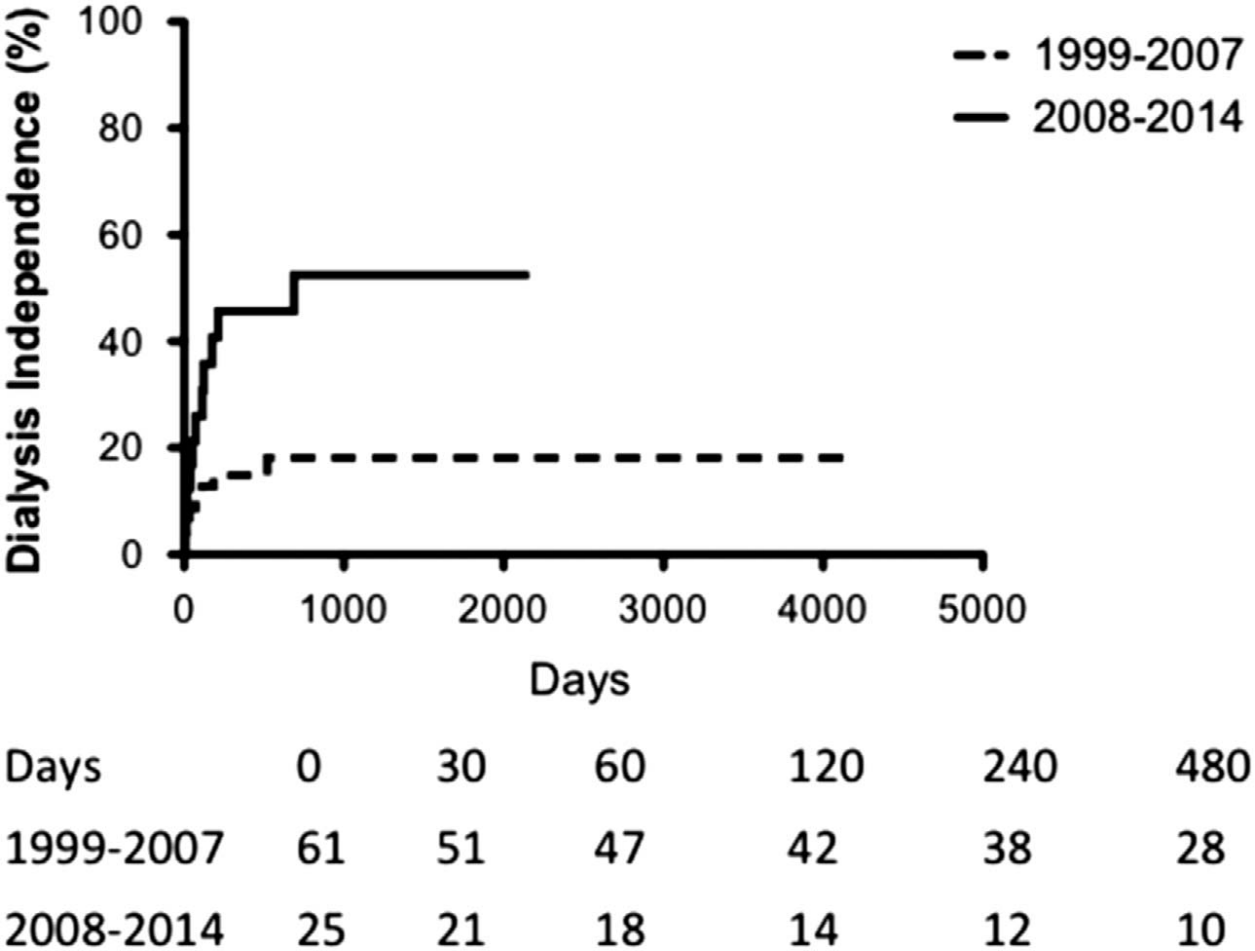
Percentage of patients reaching dialysis independence when requiring dialysis at admission. Kaplan–Meier analysis comparing ‘1999–2007' period and ‘2008–2014' period (*P*<0.05) in a log-rank comparison of the curves.

**Table 1:** Baseline clinical and biological characteristics

	*Total (*n=*135)*	*1999–2007 (*n=*88)*	*2008–2014 (*n=*47)*	P
Age (years)	68 (38–92)	70 (47–92)	67.5 (38–89)	NS
Sex ratio (M/F) %	65	63	67	NS
Dialysis dependance at diagnosis of renal involvement	86/135 (64%)	61/88 (69%)	25/47 (53%)*	<0.05
Creatinine level at diagnosis of renal involvement (μm)	499 (53–2740)	517 (53–1540)	498 (66–2740)	NS
Past history of CKD	51/135 (38%)	36/88 (41%)	15/47 (34%)	NS
Prior history of myeloma before renal involvement	19/135 (14%)	9/88 (10%)	10/47 (21%)*	<0.05
*Ig subtype*				
IgG	58/135 (43%)	37/88 (42%)	21/47 (46%)	NS
IgA	30/135 (23%)	22/88 (25%)	8/47 (17%)	NS
IgM	0/135 (0%)	—	—	
Free light chains	45/135 (33%)	28/88 (32%)	17/47 (36%)	NS
IgD	2/135 (1%)	1/88 (1%)	1/47 (1%)	NS
IgE	0/135 (0%)	—	—	
Bence Jones proteinuria (g/day)	1.8 (0.1–15)	1.5 (0.4–1.4)	2.1 (0.1–15)*	<0.05
Bone involvement of myeloma	89/135 (66%)	56/88 (63%)	33/47 (70%)	NS
Calemia (mm)	2.27 (1.4–4.6)	2.2 (1.4–4.6)	2.4 (1.5–4.4)*	<0.05
History of high blood pressure	92/135 (68%)	62/88 (70%)	30/47 (63%)	NS
Smokers	55/135 (41%)	39/88 (44%)	16/47 (34%)*	<0.05
Past history of cancer	20/135 (15%)	13/88 (15%)	7/47 (15%)	NS
Past history of cardiovascular disease (stroke, myocardial infraction, arteritis)	31/135 (23%)	22/88 (25%)	9/47 (19%)	NS

Abbreviations: CKD, chronic kidney disease; F, female; M, male; NS, not significant.

* *P*<0.05 when ‘1999–2007' is compared with ‘2008–2014'.

Comparisons between groups were performed by the Mann–Whitney *U*-test. Results are expressed as numerical variables or median (min; max).
