# Supplementary material for: Evolution in the treatment of multiple myeloma and impact on dialysis independence: data from a French cohort from 1999 to 2014
Source: Blood Cancer J. 2016 Mar 25;6(3):e409–. doi: 10.1038/bcj.2016.17 (PMC4817100; doi:10.1038/bcj.2016.17)
Supplement: Supplementary Table 1 [file bcj201617x1.docx]

|  | | 1999-2007 (n=88) | 2008-2014 (n=47) |
| --- | --- | --- | --- |
| MP | | 44/88 (50%) | 3/47 (6%) |
| Rev-Dex | | … | 3/47 (6%) |
| IVC | | 22/88 (25%) | 2/47 |
| Bortezomib containing regimens | VD | 2/88 (2%) | 19/47 (40%) |
|  | VTD | 3/88 (3%) | 3/47 (6%) |
|  | PAD | … | 1/47 (2%) |
|  | VMP | … | 6/47 (12%) |
|  | VRD | … | 6/47 (12%) |
|  | Total | 5/88 (6%) | 35/47 (74%) |
| Dexamethasone alone | | 3/88 (3%) | 0/47 (0%) |
| No CT | | 5/88 (6%) | 2/47 (4%) |
| other | | 9/88 (10%) | 1/47 (2%) |
| High dose Melphalan/ASCT | | 28/88 (32%) | 14/47 (29%) |

Supplemental Table 1 : Different chemotherapy protocols prescribed after admission for kidney involvement of multiple myeloma

MP : Melphalan-Prednison

Rev-Dex : Revlimid-Dexamethasone

IVC: Oncovin-Adriablastine-Dectancyl

Vel-Dex : Bortezomib –Dexamethasone

VTD : Bortezomib- Thalidomide – Dexamethasone

PAD : Bortezomib- Adriablastine – Dexamethasone

VMP : Bortezomib-Melphalan-Prednisone

VRD : Bortezomib- Revlimid – Dexamethasone

CT : chemotherapy

ASCT : Autologous Stem Cell Transplantation
