# Supplementary material for: Evolution in the treatment of multiple myeloma and impact on dialysis independence: data from a French cohort from 1999 to 2014
Source: Blood Cancer J. 2016 Mar 25;6(3):e409–. doi: 10.1038/bcj.2016.17 (PMC4817100; doi:10.1038/bcj.2016.17)
Supplement: Supplementary Table 2 [file bcj201617x2.docx]

|  | 1999-2007 (n=61) | 2008-2014 (n=25) |
| --- | --- | --- |
| Number of patients requiring dialysis at 30 days | 56/61 (92%) | 20/25 (80%) |
| Number of patients requiring dialysis at 6 months | 55/61 (91%) | 15/25 (60%) |
| Number of patients requiring dialysis at 2 years | 51/61 (83%) | 14/25 (56%) |

Supplemental Table 2 : Patients requiring dialysis at hospital admission
