# Supplementary figures and images for: Evolution in the treatment of multiple myeloma and impact on dialysis independence: data from a French cohort from 1999 to 2014
Source: Blood Cancer J. 2016 Mar 25;6(3):e409–. doi: 10.1038/bcj.2016.17 (PMC4817100; doi:10.1038/bcj.2016.17)

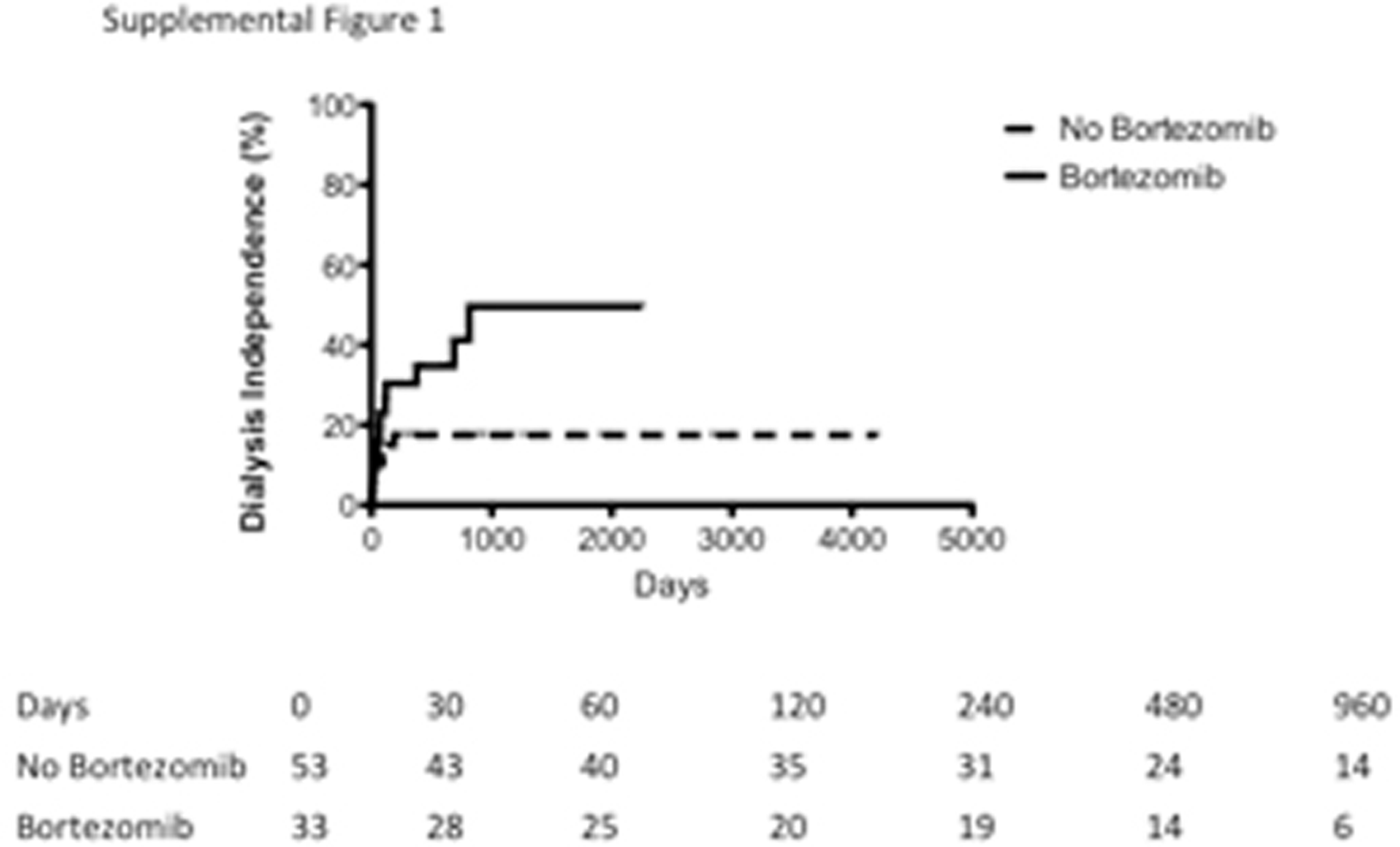

Supplement: Supplementary Figure 1 [file bcj201617x3.tif]

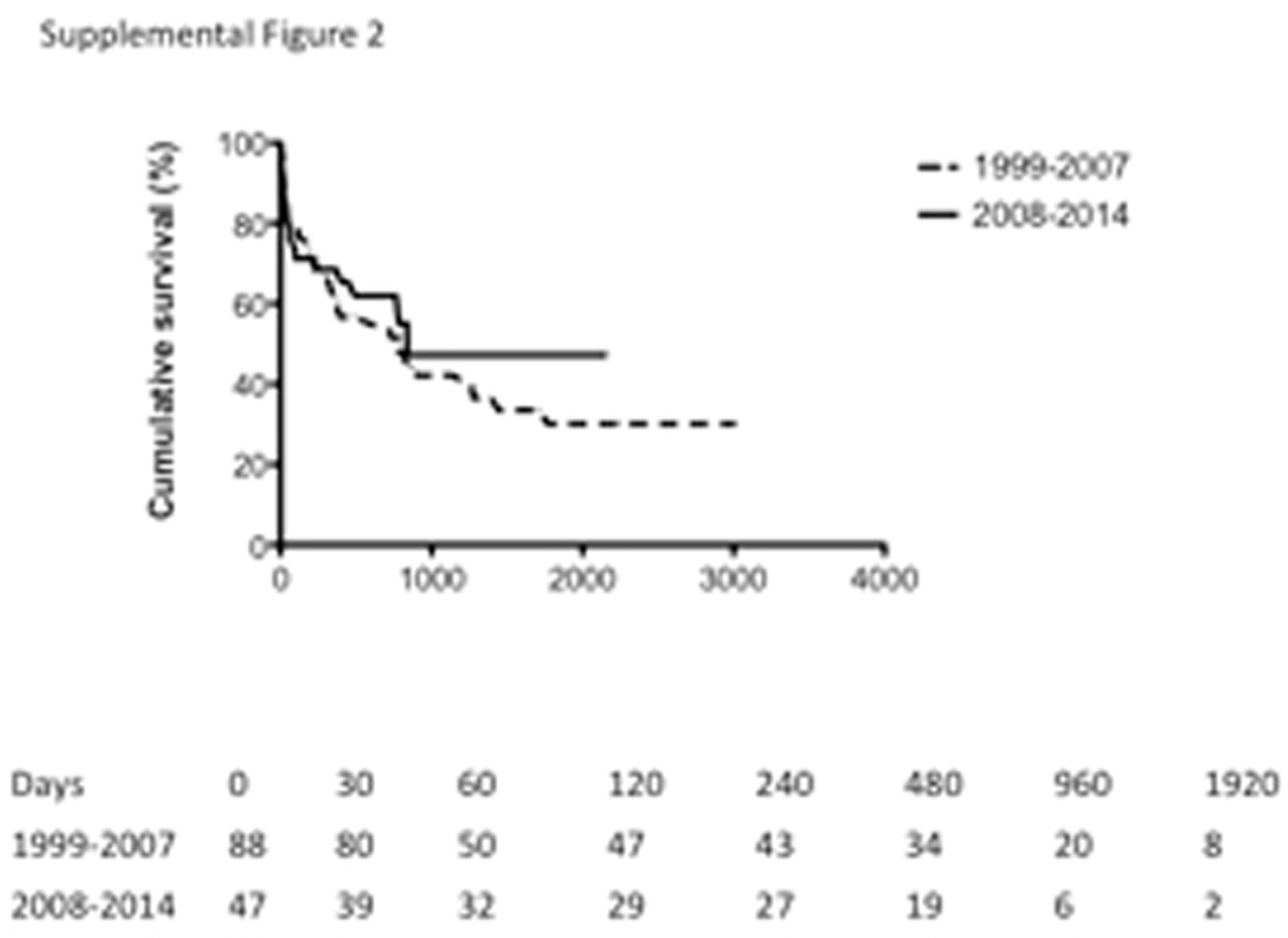

Supplement: Supplementary Figure 2 [file bcj201617x4.tif]

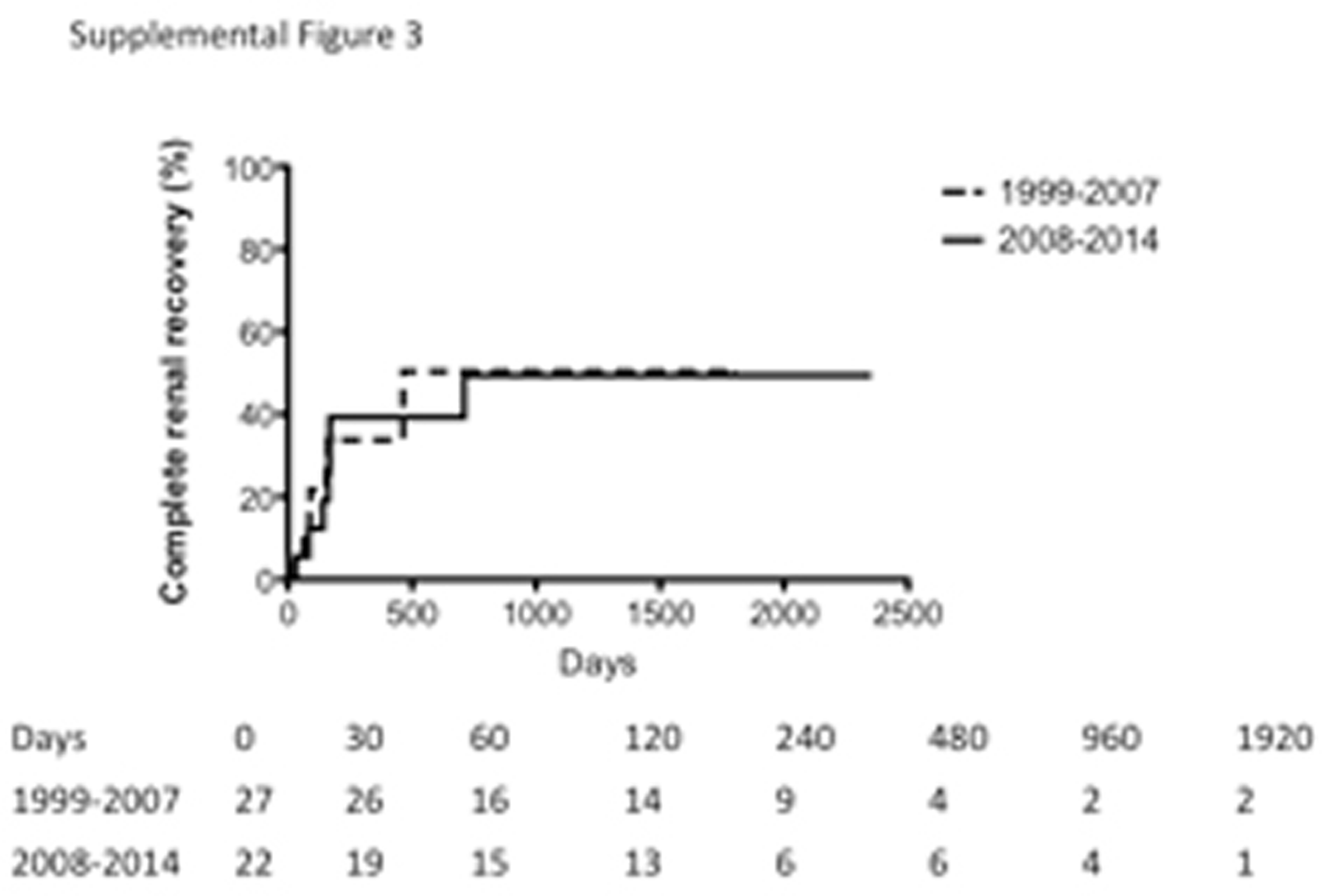

Supplement: Supplementary Figure 3 [file bcj201617x5.tif]
