# Supplementary material for: Evolution in the treatment of multiple myeloma and impact on dialysis independence: data from a French cohort from 1999 to 2014
Source: Blood Cancer J. 2016 Mar 25;6(3):e409–. doi: 10.1038/bcj.2016.17 (PMC4817100; doi:10.1038/bcj.2016.17)
Supplement: Supplementary Information [file bcj201617x6.docx]

*Supplemental Figure 1:* Percentage of patients reaching dialysis independence when requiring dialysis at admission. Kaplan Meier analysis comparing patients receiving Bortezomib versus no- Bortezomib. p<0.05 in a log-rank comparison of the curves

*Supplemental Figure 2 :*Kaplan Meier analysis estimates of cumulative survival comparing “1999-2007” period and “2008-2014” period. p=ns in a log-rank comparison of the curves

Supplemental Figure 3: Percentage of patients reaching complete renal recovery. Kaplan Meier analysis comparing “1999-2007” period and “2008-2014” period. p=ns in a log-rank comparison of the curves
